# Non-Invasive Urine-Based Diagnostic Technologies for Early Bladder Cancer

**DOI:** 10.3390/bios16030171

**Published:** 2026-03-20

**Authors:** Zhe Hao, Shuhua Yue, Lin Yao, Yanqing Gong, Jian Yu, Liqun Zhou

**Affiliations:** 1 School of Biological Science and Medical Engineering, Beihang University, Beijing 100191, China; haozhe9107@buaa.edu.cn (Z.H.); yue_shuhua@buaa.edu.cn (S.Y.); 2 National Medical Innovation Platform for Industry-Education Integration in Advanced Medical Devices (Interdiscipline of Medicine and Engineering), Beihang University, Beijing 100191, China; 3 Department of Urology, Peking University First Hospital, Beijing 100034, China; poparies@163.com (L.Y.); yqgong@bjmu.edu.cn (Y.G.); 4 Institute of Urology, Peking University, Beijing 100034, China; 5 School of Engineering Medicine, Beihang University, Beijing 100191, China; 6 Beijing Advanced Innovation Center for Biomedical Engineering, Beihang University, Beijing 100191, China

**Keywords:** bladder cancer, artificial intelligence, urinary biomarkers, multi-omics integration, non-invasive diagnostics

## Abstract

Bladder cancer (BCa) is a major global urinary tract malignancy characterized by high incidence, frequent recurrence, and significant mortality. Early diagnosis is crucial for improving prognosis and minimizing invasive procedures; however, current standard techniques, cystoscopy and urine cytology, are limited by invasiveness, cost, low sensitivity, and subjectivity. This has spurred the development of non-invasive diagnostic strategies based on urine analysis. This review highlights five emerging approaches: AI-augmented urine cytology, genomic biomarker assays (e.g., PCR and NGS for mutations and copy-number variations), DNA methylation profiling, RNA biomarkers (mRNA, miRNA, lncRNA), and protein/peptide/metabolite detection utilizing ELISA, SERS, nanozymes, and mass spectrometry. We assess the diagnostic accuracy, innovations, and clinical potential of each, while addressing persisting issues such as lack of standardization, high costs, and insufficient sensitivity for early-stage lesions. Future directions include integrating multi-omics data with AI, advancing point-of-care devices, and conducting large-scale multicenter trials. Together, these developments promise to shift BCa management toward molecular-based early detection, enabling more precise, non-invasive, and personalized patient care.

## 1. Introduction

BCa is one of the common malignant tumors worldwide. According to the latest statistics from GLOBOCAN, the number of new cases worldwide in 2022 reached 614,298, accounting for 3.1% of all cancers and causing 220,596 deaths, accounting for 2.3% of all cancer deaths [1]. According to tumor invasion depth, BCa is divided into non-muscular invasive BCa (NMIBC), which accounts for more than 75%, and muscular invasive BCa (MIBC) [2]. The main treatment method of NMIBC is transurethral bladder tumor electrolysis combined with chemotherapy and immunotherapy [3], while MIBC is mainly treated by radical cystectomy, and the quality of life of patients decreases significantly after surgery [4]. Despite improvements in surgical techniques and improved 5-year survival rates in recent years, the risk of recurrence remains high (approximately 50–70%) [5]. Furthermore, 10.4% of patients with NMIBC succumb to the disease within five years [6], whereas this figure rises dramatically to approximately 50% for those diagnosed with MIBC [7,8]. Screening of high-risk individuals can help diagnose BCa at an early stage (Ta/T1 stage), so that it can be treated more effectively [9]. Therefore, effective early diagnosis methods are crucial to reduce mortality, but there is still a lack of effective non-invasive early screening methods for BCa.

The traditional gold standard for the diagnosis and surveillance of BCa is cystoscopy combined with tissue biopsy [10]. However, this approach is invasive and is frequently associated with risks such as urethral injury and infection [10]. The procedural risks and costs are particularly elevated for patients with NMIBC who require frequent monitoring for disease recurrence [10]. Furthermore, variability in operator technique can lead to inconsistencies in the accuracy of detection [11]. Therefore, cystoscopy combined with tissue biopsy is not suitable for large-scale early screening in the general population [12]. Urine cytology represents the most convenient non-invasive diagnostic tool, with a specificity exceeding 90% [13], and thus serves as an important adjunct to cystoscopy [10]. In recent years, the urinary cytology evaluation standard “The Paris System for Reporting Urinary Cytology” (TPS) has been gradually established [14]. However, for early-stage, particularly low-grade (G1/G2) BCa, its sensitivity is significantly limited (approximately 42%) due to factors including the lack of definitive morphological atypia in malignant cells, interference from excessive inflammatory or blood cells, low cellular yield, and variability in sample preservation and processing [15,16,17,18,19,20]. Consequently, more than half of BCa cases may be missed, delaying early intervention [15]. Additionally, the diagnostic criteria and procedural protocols for conventional urine cytology are not standardized across institutions, and inter-pathologist subjectivity in assessing cellular atypia further compromises the consistency and accuracy of reports [18]. Therefore, despite its widespread clinical use, the inherent limitations of traditional urine cytology fail to meet the demands of early cancer detection.

To address these shortcomings, numerous urine-based BCa detection methods have been developed. Several have received FDA approval and been integrated into clinical practice, including assays for urinary nuclear matrix proteins (NMPs) [21,22,23,24,25], human bladder tumor antigen (BTA) [21,24,25,26], fluorescent immunohistochemistry (uCyt+/ImmunoCyt) [21,24,25], fluorescence in situ hybridization (FISH) UroVysion [21,24,27]. Although these biomarker-based tests are approved for BCa monitoring, their sensitivity and specificity for early-stage detection remain suboptimal. Moreover, conditions such as cystitis and hematuria can lead to false-positive results [21,24,27,28,29]. Thus, there is a pressing clinical need to enhance the technical capabilities of urine-based diagnostics, either by refining cytological methodologies or by developing novel urinary biomarkers with high sensitivity and specificity, to effectively meet the requirements for the early diagnosis of BCa.

**Figure 1:**
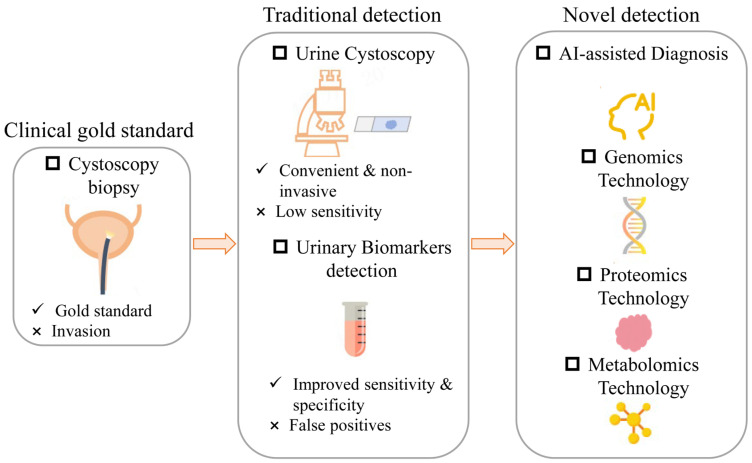
Schematic diagram of BCa detection technology development, ranging from a clinical gold standard tool, to traditional non-invasive urine detection, and to novel detection such as AI-assisted detection and multi-omics analyses.

This review will elaborate on the evolutionary trajectory of BCa detection technologies, ranging from cytology, which is the clinical gold standard, but invasive, to traditional non-invasive urine detection, including urine cytology and DNA/RNA biomarker assays, to novel detection such as AI-assisted detection and multi-omics analyses, as shown in Figure 1.

## 2. AI Empowered Urine Cytological Detection

To address the limitations of traditional urine cytology, such as low sensitivity, strong subjectivity, poor reproducibility, and time consumption, AI technology has been introduced into urine cytological detection. It has brought new breakthroughs to urine cytological detection in recent years [30]. The AI system reduces artificial errors by efficiently analyzing urine cytological images [31,32,33], automating cell-level classification [34,35] and predicting patient outcomes [36,37]. These improvements enhance diagnostic accuracy, consistency, and efficiency while optimizing diagnostic workflow [38]. Currently, the application of AI in urinary cytological detection mainly focuses on cell-level classification and patient-level diagnosis.

### 2.1. Single-Cell Level Classification

**Figure 2:**
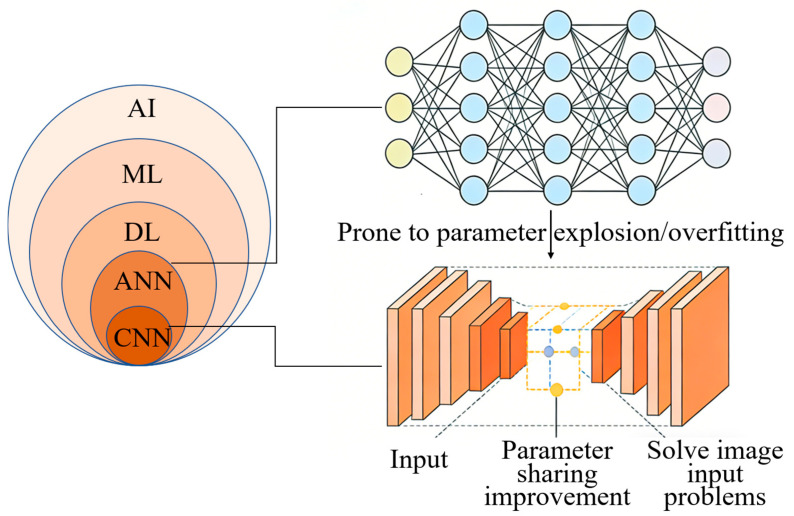
Evolution of AI in urine cytology for BCa detection, from ML to DL. Compared to the parameter-intensive and overfitting-prone ANN, CNNs employ local receptive fields, parameter sharing, and pooling. This optimizes architecture, reduces parameters, preserves spatial features, and enables automated, high-accuracy image analysis for early diagnosis. Light yellow nodes (Input layer): Input features representing clinical and biomarker data for non-invasive bladder cancer diagnosis. Light blue nodes (Hidden layers): Two fully connected hidden layers with non-linear activation functions, responsible for feature extraction and pattern recognition. Light pink and light purple nodes (Output layer): Output nodes representing the diagnostic classification results (e.g., benign/malignant, or risk stratification). AI—Artificial Intelligence; ML—Machine Learning; DL—Deep Learning; ANN—artificial neural networks; CNN—convolutional neural network.

In 2015, the DL model based on artificial neural networks (ANNs) was first applied to urine cytological detection [33]. ANN is a computational model inspired by biological neurons, consisting of multi-layer interconnected artificial neurons that achieve nonlinear data mapping by adjusting synaptic weights and activation functions, thus completing pattern recognition [39,40]. Its training process is based on a backpropagation algorithm, which uses gradient descent to optimize the loss function to minimize prediction errors, and extracts hierarchical features of data through hidden layers [41]. However, the ANN model with a fully connected structure cannot take the image directly as input, which is prone to parameter explosion and overfitting problems [40]. A multi-layer convolutional neural network (CNN) optimizes the network structure and extraction method of input features based on ANNs [42], as shown in Figure 2. It efficiently captures spatial local features (such as textures and edges) through the local receptive field, parameter sharing, and pooling operations of the convolution layer. Thus, it significantly reduces the number of parameters while retaining the topological structure of the input data [40]. A multi-layer CNN model using 2405 urine cytological full-slide images as input achieved an area under the curve (AUC) of 0.88 in the diagnosis of high-grade BCa, with a sensitivity of 79.5% and a specificity of 84.5% [32]. The issues of parameter explosion and overfitting problems caused by directly adaption of images as input in the ANN model are solved.

To improve the interpretability of the model, it is necessary to identify urinary cell types. A study enhanced the accuracy of urinary cell classification by a fine-grained CNN model, as shown in Figure 2. They reached an AUC of 0.989 in the detection of high-grade urothelial cancer cells, with an accuracy rate of 95.36% [34]. Wu et al. used a multi-scale feature fusion strategy to eliminate background noise interference, thereby improving the accuracy of urinary cell segmentation [43]. In addition, by combining different types of cell morphological characteristics with CNN, such as nucleoplasm ratio, nuclear area, and chromatin atypicality, the sensitivity to recognition of malignant tumor cells reached 0.896–1.000. To improve cell segmentation accuracy, Kaneko et al. reduced boundary artifacts based on the superimposed segmentation tool named “CutMix” and adjusted the weight of the label [31]. A cell segmentation tool named “CircleCut” that handles irregular cell boundaries was developed by using 4637 cell annotation images. Combining with refined feature data augmentation technology and CNN, the cell classification model training was completed. The AUC of urinary cell classification reached 0.99, with an accuracy rate of 95%, a sensitivity of 96.7%, and a specificity of 95%. AI classification based on urine cytology improves the accuracy and efficiency of diagnosis, providing a solid foundation for the development of an AI-based urine cytological diagnostic platform.

### 2.2. Patient-Level Diagnostics

The deep learning system based on CNNs can effectively predict the existence of malignant tissue in urine cytopathological images, and its prediction is interpretable based on the abnormal cells selected by the model [44]. The fully automatic AI pathological prediction system was achieved by analyzing digitized urinary cytology images and combining semi-supervised learning methods. In the test of 315 digitized urine cytology slides, with a comparable specificity and a consistency of 86%, this AI system achieved significantly higher sensitivity (63%) than a cytopathologist (46%) [45]. The latest research also shows that the application of AI in pathological prediction is not limited to the field of cytology. The Virchow model is by far the largest fundamental model of computational pathology, which can achieve an AUC of 0.95 at the individual level in nine common cancers and seven rare cancers [46]. This demonstrates its outstanding performance in pan-cancer detection. And, it shows that AI technology has broad application and great potential in the field of pathological prediction.

Based on AI segmentation and classification of urinary cells, a fully automated BCa detection platform based on urine cytology named VisioCyt has been developed. This approach enabled the differentiation of urothelial cells, squamous cells, and inflammatory cells. In a multicenter prospective study, the assay achieved a sensitivity of 84.9%; notably, the sensitivity for low-grade tumors reached 77%, which was substantially higher than the 26.3% observed with conventional urine cytology in this study [47]. Recently, a commercial BCa AI detection platform based on urine cytology named AIxURO has been developed, which is used to identify different types of cells in urinary cytology images and provide quantitative analysis. The AIxURO platform integrates the morphological diagnostic criteria from TPS 2.0 [14], including nucleoplasm ratio, nuclear area, and chromatin abnormal characteristics, thereby enabling high-throughput digital analysis of urothelial cells. Based on the contrast learning framework, its multi-scale feature fusion mechanism can eliminate background noise interference and optimize diagnostic specificity. The AIxURO platform-assisted diagnosis achieved a sensitivity of 92.3% and a specificity of 100%, with a high concordance with expert consensus. Specifically, an average of 686 abnormal cells were identified in positive cases, whereas only eight abnormal cells were detected in negative cases [48]. Combined with an anchor quality sampling strategy and embedding model, the AIxURO platform’s recognition sensitivity to a typical urothelial cell was increased from 25.0% to 63.9%, while significantly shortening the screening time [37]. For postoperative follow-up of upper urinary tract urothelial carcinoma (UTUC), the AIxURO platform increased the detection rate of early recurrence from 29.6% (8/27) to 37.0% (10/27), enhancing the credibility of urinary cytology reports and helping to detect intravesical recurrence in the early stage [36].

Conventional urine cytology is limited by inadequate diagnostic sensitivity when it comes to early-stage and low-grade BCa. In contrast, AI technologies, especially those based on CNNs, can markedly boost diagnostic performance through automated cell segmentation, precise classification, and quantitative morphological analysis. These AI systems excel at identifying subtle cytological abnormalities, such as elevated nucle-ar-to-cytoplasmic ratios, enlarged nuclear areas, and chromatin irregularities, which in turn enables highly sensitive detection of early-stage tumors. A case in point is the Visio-Cyt platform, which has raised the sensitivity for low-grade BCa detection from 26.3% (achieved by conventional urine cytology) to 77%. In summary, by enhancing the capture and interpretation of early biological signals, AI has transformed urine cytology into a highly sensitive, non-invasive tool for screening and monitoring, holding significant promise for the early diagnosis and postoperative surveillance of BCa.

## 3. Detection Technologies of Urine Genome Technology

Urine samples of BCa patients contain a large amount of tumor-derived DNA/RNA, which can be used as liquid biomarkers for BC diagnosis and monitoring [49,50,51]. The development of genetic detection technology for early BCa diagnosis mainly focuses on “improving the detection rate of small tumors” and “breaking through early missed bottlenecks”. Complementary systems are formed through targeted optimization and multi-dimensional integration of different technologies.

### 3.1. Detection Technologies of Urine DNA

#### 3.1.1. Polymerase Chain Reaction (PCR) Detection Technology

The PCR technique provides a foundation for precise detection based on gene expression. Based on this, the two-gene ratio model (with an upregulated gene as the numerator and a downregulated gene as the denominator) represents a significant advancement in the development of early diagnostic methods. A study established a DNA discriminant score based on the urinary ratios of IQGAP3/BMP4 and IQGAP3/FAM107A [52]. This model achieved an AUC of 0.862 for the diagnosis of early-stage, particularly low-grade BCa, with a sensitivity of 71.0% and specificity of 88.6%. This study provides the first evidence that gene ratio analysis can effectively differentiate between early-stage cancer and hematuria, laying the foundation for the subsequent application of PCR technology in NMIBC. Moreover, the IQGAP3/BMP4 ratio is strongly correlated with the recurrence-free survival in patients with NMIBC, suggesting that this index cannot only be used for early diagnosis, but also predict the risk of recurrence 1–2 years post-surgery [53].

Based on previous studies on BCa DNA mutations, telomerase reverse transcriptase (TERT) gene promoter mutations [54,55], and fibroblast growth factor receptor 3 (FGFR3) mutation [56,57] have been the most common somatic mutation. By integrating the two-gene model and orthodenticle homeobox 1 (OTX1) gene mutation detection, a three-gene urine assay for early BCa detection was developed [58,59]. In a prospective multicenter trial, this assay achieved a sensitivity of 57% for recurrence monitoring of early BCa, covering 80% of early recurrence cases [60]. And, it further proved that “multi-gene collaboration” is better than single-gene detection, thereby providing ideas for missed diagnosis and prevention of early-stage BCa. This idea directly inspired the development of Uromonitor^®^ detection [61]. To further enhance diagnostic sensitivity, OTX1 gene mutation detection was replaced with the KRAS gene mutation-based Uromonitor^®^ detection [61,62], which increased the overall sensitivity for BCa recurrence to 100% (although the sample size needs to be considered). Especially for low-grade tumors, it has a significantly higher sensitivity (65%) than urinary cytology (0%) in this study [61]. For the detection of early recurrence with 6–12 months postoperatively, this assay achieved a sensitivity of 93.1% and a specificity of 85.4%, while enabling the detection of small tumors (diameter <5 mm) [63], while the sensitivity of urine cytology for early postoperative recurrence in this study was only 26.3%. This solved the problem of insufficient sensitivity of the OTX1 gene mutation in early multi-gene combination detection. In addition, the negative predictive value (NPV) of the Uromonitor^®^ test is as high as 98.8% [64]. This robust NPV supports the safe reduction in cystoscopy frequency by approximately 30%, especially suitable for non-invasive long-term follow-up of patients with early BCa.

PCR technology has become the first choice for early screening due to its fast and low-cost characteristics. In particular, Uromonitor^®^ with high NPV (>95%) can effectively eliminate early recurrence and reduce invasive examinations; its limitation is that it has insufficient sensitivity to small tumors (about 60%), and it depends on sequencing technology to supplement it.

#### 3.1.2. Next-Generation Sequencing (NGS) Technology

NGS technology breaks through the bottleneck of small-tumor detection through multi-dimensional molecular characteristics. It can perform rapid and high-throughput sequencing of large amounts of DNA or RNA at the same time [65], with workflow involving library construction, template preparation, sequencing reactions, and bioinformatic data analysis [66]. And, it is divided into whole-genome sequencing (WGS) technology [67] and deep sequencing technology [68].

WGS is an important application of NGS, which performs comprehensive and non-selective sequencing of the entire genome of an organism [67]. Based on WGS technology, it was found that mutations of 11 genes in urine and copy number variations (CNVs) of 39 chromosomal arms were related to BCa. And the UroSEEK detection scheme was developed with a sensitivity of 67% for low-grade BCa detection, far exceeding urinary cytology (0%) in this study. It can detect early tumors containing only 1–2 gene mutations, and effectively identify small tumors missed by traditional methods [69], solving the problem of “high missed detection rate” by PCR technology in low-grade tumors. Moreover, it is confirmed that the frequency of TERT promoter mutations in low-level non-invasive BCa is as high as 77% [70], becoming the core detection target of this technology. However, WGS technology is expensive, limiting its widespread use. To address this issue, shallow whole-genome sequencing (sWGS) was introduced, reducing per-base coverage while maintaining genome-wide information through imputation algorithms and population genetics modeling [71,72,73]. This approach cuts sequencing costs to ~20% of WGS while preserving high-resolution variant detection, especially in large cohorts (>2000 samples) [72]. Based on sWGS technology, a diagnostic model named UCdetector was developed [74]. The UCdetector model was sensitive to the early stage with a specificity of 94.7%, and especially for small-tumor detection rate, which reached 78%, solving the limitation of PCR technology relying on “high abundance mutations”. With the joint development of sequencing technology and AI technology, the UCseek technology was developed, and it had good performance at aWGS sequencing depths of 0.3×–0.5× [75]. The detection accuracy for low-grade and early-stage tumors is 91.8% and 94.3%, respectively. The sensitivity of monitoring recurrence is 90.91%, which was significantly better than cystoscopic imaging (59.09%), proving that gene mutation analysis can improve the detection rate of small tumors and become a potential tool for early screening in grassroots hospitals. When the sequencing depth is further reduced to 0.1×, low-coverage WGS (LC-WGS) was developed, and the sequencing cost was further reduced. The sensitivity to early-stage BCa detection was 82.5%, specificity was 96.9%, and the AUC in the T1 stage of BCa reached 0.91 [76], which was suitable for large-scale early screening.

Another development direction of NGS technology is to obtain more accurate genomic information, so deep sequencing technology has been developed [77]. By performing multiple independent sequencing reactions on DNA or RNA fragments in the same region, false-positive results due to random errors are reduced, and detection sensitivity and accuracy are improved [78]. During data analysis, specific algorithms and statistical models [77] are employed to integrate and interpret the repeated sequencing data, thereby enabling the more reliable identification of low-frequency mutations, rare variants, and complex gene expression patterns. It is particularly valuable in studying tumor heterogeneity [79], pathogen mutation [80], and evolution [81,82]. For patients with hematuria, an ultra-deep sequencing (23 genes and 443 mutations) yields a sensitivity of 87.3% and specificity of 84.8% for the detection of early-stage disease. Additionally, this assay achieved a sensitivity of 86.2% for the NMIBC recurrence, with the false-positive rate controlled at 15.2% [83]. uCAPP-Seq technology also has a good effect in predicting the applicability of immunotherapy [84], extending deep sequencing from “early diagnosis” to “prognostic stratification” for the first time [85]. To detect residual disease with high sensitivity, uCAPP-Seq technology was employed to integrate ultra-low throughput WGS (ULP-WGS) with personalized profiling of BCa. Using leave-one-out cross-validation, a random forest model incorporating urine cell-free DNA (cfDNA)-derived factors yielded 87% sensitivity in predicting residual disease. This proved the superiority of urinary multitopic analysis in tumor residue detection and provides a molecular basis for personalized treatment decisions.

PCR techniques have emerged as pivotal tools for initial screening, capitalizing on their inherent advantages of rapidity and cost-efficiency. Meanwhile, NGS technologies have further elevated the detection sensitivity for low-grade and small tumor lesions—with some assays achieving sensitivity exceeding 90%—by integrating multidimensional molecular features such as gene mutations and CNVs. The technological advancement in this field has followed two primary directions: on one hand, reducing costs via shallow or low-coverage sequencing to expand applicability in primary healthcare settings; on the other hand, leveraging ultra-deep sequencing and multi-omics integration to realize high-sensitivity detection and prognostic stratification. Collectively, these innovations are redefining urine-based testing from a supplementary approach to a highly sensitive, standardized tool for early screening and recurrence monitoring, thereby furnishing critical support for the precision diagnosis and comprehensive management of BCa.

### 3.2. Urine DNA Methylation Detection Technologies

#### 3.2.1. Methylation-Specific PCR (MSP) Technology

With its high sensitivity and specificity [86], MSP technology based on PCR technology has become the basic platform for urine methylation detection [87,88]. By targeting the methylation status of key genes, it has built two-marker or multi-marker models that have been developed to overcome the limitations of traditional methods in early BCa, especially low-grade NMIBC detection. Through MSP technology, the methylation level of DMRTA2 showed higher sensitivity in the early stage of detection, with T1 at 94.1%, T2 at 96.4%, T3 at 77.8%, and T4 at 71.4% [89]. MSP technology detects six methylation markers (ASTN1, DLX1, etc.) to build a GynTect test solution, with 60% sensitivity and 96.7% specificity in NMIBC detection. The sensitivity can be increased to more than 90% through algorithm optimization [88], confirming the potential of MSP technology in low-grade tumor screening. Reduce six methylation markers to four (HOXA9, PCDH17, POU4F2, and ONECUT2), combined with a high-resolution melting curve, detection sensitivity reaches 90.5%, and PPV reaches 100% [90]. Through bioinformatics-driven optimization, the panel was reduced to two markers, improving sensitivity to 91.2%. For early-stage BCa, MSP demonstrated superior sensitivity compared with urine cytology (88.1% vs. 55.6%) and FISH (89.7% vs. 72.2%). Importantly, its application could reduce unnecessary cystoscopies by approximately 30% [91], verifying the efficient detection ability of MSP technology for early BCa.

**Figure 3:**
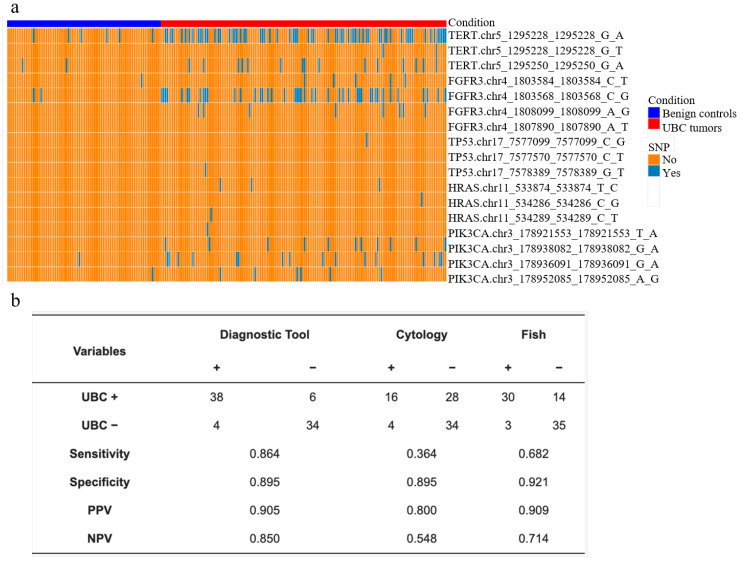
Validation of the biomarker as a diagnostic tool, external validation of the model, and comparison with FISH and cytology. (**a**) The heatmap of different single-nucleotide polymorphism sites. The parameter “condition” represents the benign controls in blue and the BCa tumor samples in red; (**b**) The diagnosis performance of different techniques. Figure reproduced with permission from ref. [93]. Copyright from 2023, MDPI Limited.

However, single-marker assays show limited sensitivity across different BCa stages, indicating the need for the combined use of different markers. Through methylation detection and gene mutation detection, the AUC of different stages was 0.96, sensitivity was 93%, specificity was 86%, and NPV was as high as 99%, indicating that 77% reduction in unnecessary cystoscopy [92]. Later, NGS technology was introduced to combine with MSP, and it was found that the three markers, a combination of NRN1 methylation combined with TERT and FGFR3 mutations, showed good performance in differentiating BCa from controls (Figure 3a). In external verification, comparing with cytology and FISH, as shown in Figure 3b, the model was achieved with a superior sensitivity of 86.4% and a comparable specificity of 89.5% [93]. MSP technology can accurately capture early tumors by targeting methylation sites of known cancer-related genes (such as TWIST1, RUNX3, HOXA9). Especially in NMIBC monitoring, the EpiCheck series of studies has shown that its NPV is as high as 95.1%~99.3%, which can reduce unnecessary microscopy by more than 80% [94,95,96,97,98,99,100]. Its core advantage is its simplicity of operation, high throughput, and suitability for routine clinical screening. Multi-marker combinations (such as the five-marker stratification model) further enhance the recognition ability of high-risk NMIBC and MIBC (sensitivity 90.5%, specificity 86.8%), providing candidate markers for subsequent in-depth analysis at the genomic level.

#### 3.2.2. DNA Methylation Detection Technology

By high-throughput analyzing multiple gene methylation patterns, combined with mutation status and clinical parameters, it constructs more complex prediction models, demonstrating high application value in early diagnosis, recurrence monitoring, and prognosis evaluation.

A study screened three biomarkers (a combination of hypermethylation and hypomethylation) using pyrosequencing. The AUC for predicting NMIBC recurrence reached 0.95, with a sensitivity of 89% and specificity of 97%, significantly outperforming cytology (35%) and cystoscopy (15%) [101]. This study first confirmed the predictive efficacy of dynamic methylation changes for recurrence risk. Subsequently, another study found that hypermethylation of the HIST1H4F gene was significantly increased in BCa [102]. Although not directly associated with clinicopathological parameters, its stable methylation status provides a new candidate biomarker for early screening. Meanwhile, a separate study combined SNaPshot methylation analysis with gene mutation detection to construct a prediction model incorporating age factors [58]. The NPV reached 99.6%~99.9% in hematuria patients, indicating an extremely low false-negative rate. Later, a study reported that the combination of EpiCheck detection and urine cytology achieved a sensitivity of 83.3% and specificity of 86.3% in monitoring high-grade BCa, confirming that methylation biomarkers can compensate for the missed diagnosis of low-grade tumors by cytology [97]. More recently, a study developed an eight-biomarker model (FGFR3 and TERT mutations + OTX1 and ONECUT2 methylation) based on a cohort of high-risk hematuria patients following AUA guidelines [103]. The AUC ranged from 0.929 to 0.971, and the probability of BCa in negative cases of the low-risk group was only 0.3%~2%, enabling safe avoidance of cystoscopy, while positive cases in the medium–high risk group required accelerated evaluation to achieve precise stratification. The success of such models relies on the analysis of methylation regulation of key signaling pathways (e.g., Wnt and Notch pathway-related genes) in BCa initiation and progression, providing a basis for personalized diagnosis and treatment.

#### 3.2.3. DNA NGS Technology

NGS technology enables unbiased scanning of genome-wide methylation patterns, identifying differentially methylated regions (DMRs) and tissue-specific biomarkers, and has achieved breakthroughs in tumor grading, localization, and ultra-sensitive detection, serving as a bridge connecting basic research and clinical applications.

A study identified methylation signatures of 32 genes (e.g., EOMES, GP5, ZSCAN12) using microarray-based whole-genome bisulfite sequencing (WGBS), which could completely distinguish low-grade from high-grade BCa [104]. Among these, GP5 and ZSCAN12 were newly discovered high-grade specific hypermethylated genes, providing molecular biomarkers for pathological grading. Another study developed the GUseek system, which integrated methylation and CNVs through shallow WGBS [105], its accuracy exceeded 95% in detecting genitourinary system cancers, with a sensitivity of over 72% for distinguishing BCa, prostate cancer, and renal cell carcinoma, realizing “one-stop” non-invasive localization. However, in terms of the practical application of this technology, there are still real obstacles, such as the high cost of WGBS for batch samples, the high cost of sequencing instruments, and the complex sample pretreatment. Additionally, the UroMark test, based on NGS analysis of 150 CpG sites, achieved an AUC of 97%, sensitivity of 98%, specificity of 97%, and NPV of 97% in the validation cohort, becoming the first genome-wide methylation detection protocol approaching clinical application standards [106]. Moreover, the UCseek technology achieved an accuracy of 94.3% for early UC at an ultra-low sequencing depth (0.3×–0.5×), with a recurrence monitoring sensitivity of 90.91%, far exceeding cystoscopy (59.09%) [75]. This confirmed that genome-wide methylation signatures can be stably detected in trace amounts of DNA, enabling rapid outpatient testing.

In addition, a study identified fragmentomics-based methylation analysis (FRAGMA) technology, which enabled the inference of methylation status through cfDNA fragmentation patterns without bisulfite treatment, achieving an AUC of 0.98 in the diagnosis of hepatocellular carcinoma and nasopharyngeal carcinoma [107]. This provides a new methodological approach for cfDNA methylation detection in BCa, especially suitable for analyzing trace cell-free DNA released by small tumors. It can solve the problem of traditional detection’s dependence on sample volume.

From PCR to whole-genome sequencing, urinary DNA methylation detection of BCa has gradually advanced from “targeted validation” to “panoramic analysis”. Due to its high sensitivity and operability, PCR technology remains the mainstay for clinical translation; genomic sequencing technology improves the detection rate of small tumors through multi-omics integration; whole-genome sequencing technology lays the foundation for precision medicine. In the future, for further development of early BCa diagnosis, further optimization of biomarker combinations, reduction in sequencing costs, and validation of their clinical application through large-scale multicenter prospective studies are necessary.

### 3.3. Urine RNA Detection Technologies

#### 3.3.1. mRNA Detection Technology

The establishment of standardized detection workflows has laid the foundation for reliable analysis of urine mRNA biomarkers. However, early studies were hindered by sample heterogeneity and donor variability. In 2007, a study first introduced a standardized workflow with external RNA controls (RNAₗᵤC), in which RNA from whole urine, cell pellets, and cell-free fractions were analyzed via RT-qPCR to address the interference of urine components [108]. The ETS2/uPA mRNA ratio in this study achieved a whole-urine detection of BCa with specificity of 100% and AUC of 0.929, which is better than the traditional tissue marker hTERT. However, its sensitivity for low-grade tumors was only 53.9%, indicating that single biomarkers have limited ability for the detection of early small tumors, which requires technical innovation and combination optimization. This standardized technology provided a methodological template for future research, promoting the development of urine mRNA detection from experimental exploration to clinical translation.

With the development of high-throughput technologies, researchers have shifted from single-gene analysis to multi-gene combination modeling. A study quantified 45 genes using TaqMan Arrays and verified the superiority of the GS_D2 model (IGF2 and MAGEA3), which achieved an AUC of 0.918 and a sensitivity of 94.74% for high-risk tumors with strong cross-center stability [109]. However, its sensitivity for low-risk tumors was 67.86%, which still needs improvement. In parallel, another study focused on carbonic anhydrase IX (CAIX) splice variants and found that the proportion of full-length isoforms (FL%) was significantly increased in cancer patients (median 70.8% vs. 2.6% in controls) with an AUC of 0.837 and sensitivity of 90%, providing a new dimension of “splice variant ratio” for biomarker screening [110]. Furthermore, a study discovered that NDRG2 mRNA was significantly downregulated in the urine of BCa patients [111]. Combined with protein level verification, the AUC reached 0.888 with a sensitivity of 85.5%, and it was associated with tumor grade and stage, demonstrating the clinical value of in-depth single-gene mining.

In the exploration of clinical utility, a study demonstrated through Cxbladder Monitor in 2017 that gene expression analysis combined with clinical data can construct an efficient “rule-out” detection model, achieving a sensitivity of 91% and NPV of 96%, with a sensitivity of 84% for low-grade tumors, significantly reducing unnecessary cystoscopies [112]. Building on this, a study developed an 8-gene classifier (including ANXA10 and IGF2) optimized for follow-up scenarios, achieving a sensitivity of 94% and NPV of 98% for low-risk NMIBC, which could reduce invasive procedures by 17% [113]. Later, a machine learning (ML)-based UriBLAD model (32 genes) further broke through performance bottlenecks, with an AUC of 0.93 in the validation cohort and a sensitivity exceeding 80% for both non-muscle-invasive and low-grade cancers, demonstrating the strong generalization ability of multi-omics data and algorithm integration [114].

#### 3.3.2. miRNA Detection Technology

As key molecules regulating gene expression, miRNAs are stably present in urine, and early studies screened differential molecules through microarray chips combined with qPCR verification. A study identified 15 differential miRNAs in BCa tissues, among which four (e.g., miR-141 and miR-205) could distinguish NMIBC from MIBC, with a combined detection accuracy of >75%. This study was the first to demonstrate that miRNA expression profiles possess both diagnostic and prognostic value [115]. In the same vein, another study found that urinary miR-1255b-5p had a sensitivity of 85% for invasive cancer, initiating the exploration of miRNA detection in non-invasive samples and establishing the classic “chip screening-qPCR verification” workflow [116].

NGS has promoted miRNA detection into the genome-wide era. A study screened three core miRNAs (e.g., let-7c-5p and miR-30a-5p) through NGS, and the combined model achieved 0.70 of AUC in distinguishing BCa from controls while being able to identify tumor grade and invasiveness [117]. To address the low miRNA content in early small tumors, a study combined telomerase extension reaction with graphene oxide (GO) signal amplification in 2017 to construct a combined detection method for proteins and miRNAs [118]. The five selected miRNAs (e.g., let-7b-5p) significantly improved the discrimination efficiency between NMIBC and MIBC. Innovatively, a study innovatively integrated miRNAs (e.g., miR-34a-5p) with surface-enhanced Raman scattering (SERS) and constructed a combined model using ML, achieving an AUC of 0.92 in distinguishing BCa from controls and 0.95 in distinguishing molecular subtypes, providing a new strategy for point-of-care rapid diagnosis [119].

In addition, miRNAs in urinary extracellular vesicles (EVs) are more stable due to vesicle protection. A study identified miR-21-5p from urinary EVs, which achieved a diagnostic sensitivity of 75.0% and specificity of 95.8%, with a sensitivity of 75.0% even in urine cytology-negative patients [120]. Its expression significantly decreased after surgery, demonstrating the advantage of EVs as biomarker carriers. Another study established a 6-miRNA signature (including let-7c and miR-135a) through TaqMan microarrays, achieving an overall diagnostic AUC of 88.3% and an AUC of 92.9% for high-grade NMIBC, which was not interfered with by clinical factors [121]. A meta-analysis showed that the combined AUC of urinary miRNAs was 0.88, and multi-miRNA combinations were superior to single biomarkers, among which miR-143 performed prominently (AUC 0.88), verifying the universal advantage of combined models [122].

#### 3.3.3. lncRNA Detection Technology

Long non-coding RNAs (lncRNAs) have become emerging targets for non-invasive detection due to their long sequences and high stability. A study developed a gold nanoparticle (Au-NP) hybridization method for detecting lncRNA-UCA1 without PCR, which could complete detection within 30 min through color change, achieving a sensitivity of 92.1% and specificity of 93.3%, with a consistency of 98% with qRT-PCR [123]. It is particularly suitable for early screening in schistosomiasis-endemic areas, addressing the demand for convenient detection in primary medical care.

Based on qRT-PCR quantitative analysis and microarray screening, a study screened a two-lncRNA combination (uc004cox.4 upregulation and GAS5 downregulation). The validation cohort achieved an AUC of 0.885, sensitivity of 84.5%, and specificity of 78.2%, significantly outperforming urine cytology [124]. High expression of uc004cox.4 was associated with NMIBC recurrence. Another meta-analysis showed that the overall combined AUC of urinary lncRNAs was 0.86, and intracellular lncRNAs (e.g., UCA1) had better performance than free forms due to stability advantages. Multi-lncRNA combinations (AUC 0.87) were slightly superior to single indicators (0.85), confirming core biomarkers such as UCA1 and H19 [125].

Based on bioinformatics analysis of genomic instability, a study constructed a 5-lncRNA signature (GllncSig), which was associated with overall survival and chemotherapy sensitivity of BCa. The low-risk group was more sensitive to drugs such as cisplatin (lower IC50) [126]. Although this study was based on tissue samples, the screened molecules (e.g., CFAP58-DT and MIR100HG) provide potential targets for urinary biomarker combinations, promoting the extension of lncRNA detection from simple diagnosis to “diagnosis–prosmallgnosis-treatment” whole-process management.

Urine RNA detection technology has achieved significant progress in early BCa diagnosis, especially in the detection of tumors and NMIBC, through mRNA standardization and multi-gene modeling, miRNA high-throughput screening and signal amplification, and lncRNA nano-visualization and combination optimization. Each of the three types of RNA technologies has unique advantages: mRNA biomarkers (e.g., ETS2/uPA, IGF2/MAGEA3) have shown high specificity in clinical validation, suitable for rule-out detection; miRNAs (e.g., miR-21-5p, 6-miRNA signature) rely on their stability and multi-modal combination to improve the capture ability of trace tumors; lncRNAs (e.g., UCA1, uc004cox.4 + GAS5) have expanded convenient detection and prognostic prediction scenarios through nanotechnology and bioinformatics. However, current technologies still face common challenges: insufficient standardization, as differences in sample processing, internal reference selection, and detection platforms among different studies lead to result heterogeneity, requiring the establishment of unified technical specifications; early sensitivity bottlenecks, as the molecular enrichment efficiency for extremely early micro-tumors such as carcinoma in situ still needs improvement, requiring the analysis of tumor heterogeneity through single-cell sequencing and spatial transcriptomics; the need for breakthroughs in multi-omics integration. Currently, the three types of RNA technologies are being developed independently. In the future, it is necessary to construct mRNA-miRNA-lncRNA combined models, combined with DNA methylation, protein biomarkers, and clinical parameters, to form a multi-dimensional diagnostic system.

Although multi-omics and NGS technologies have powerful analytical capabilities in identifying genomic biomarkers (DNA/RNA) and improving diagnostic accuracy, their widespread application still has limitations. Despite considerable efforts, the testing costs remain relatively high. For example, a single multi-gene test (such as Uromonitor) is much higher than conventional urinary cytology tests. This cost gap may limit the broad adoption of these technologies in routine primary care, especially in low- and middle-income regions with limited medical resources. In addition, the integration of multi-omics and NGS technologies requires additional investment in equipment, professional technical training, and data management systems, which further increases the economic burden on primary healthcare institutions. However, it is important to note that long-term economic benefits may outweigh the initial high investment. For example, the high NPV of multi-gene testing can effectively reduce the frequency of cystoscopy, thereby lowering the overall medical costs for patients suspected of having bladder cancer. Therefore, from a long-term perspective, if the equipment costs of these technologies can be reduced and their procedures standardized, incorporating them into routine primary healthcare may be economically feasible.

## 4. Protein/Peptide Biomarker Detection Technology

Protein/peptide biomarkers have become core targets for non-invasive BCa detection due to their strong functional correlation with tumor initiation and invasion (e.g., NMP22, BTA, surviving). Enzyme-Linked Immunosorbent Assay (ELISA) is a widely used non-invasive technique for urinary biomarker detection in BCa, facilitating early screening, recurrence monitoring, and prognostic assessment via quantifying biomarkers such as nuclear matrix protein 22 (NMP22) [127], surviving [128], and vascular endothelial growth factor (VEGF) [129]. But its clinical utility is constrained by analytical challenges, highlighting the need for assay optimization and standardization to enhance accuracy in precision BCa management. Core breakthroughs in biomarker detection technologies have focused on three directions: low-abundance protein capture, signal amplification, and multi-biomarker integration. Over the past 5 years, they have shown a convergent innovation trend of “nanotechnology + AI”.

### 4.1. Proteomic Technologies

A series of studies have continuously promoted its clinical application value, gradually achieving breakthroughs mainly in diagnostic accuracy and standardization. In particular, a classic study of multicenter external validation laid a foundation for the clinical application of multiplex urinary protein panels [130]. This study validated the panel in a large cohort across multiple institutions, demonstrating robust diagnostic performance with an AUC of 0.87 for BCa detection, particularly excellent sensitivity (83%) for early-stage tumors, and good reproducibility across different clinical laboratories, which is crucial for promoting the standardized application of proteomic assays in clinical practice [130]. Further clinical validation of multiplex protein assays was subsequently conducted. A 10-plex urinary protein assay (Oncuria assay) targeting 10 key analytes (including angiogenin, IL-8, and VEGF-A) was clinically validated, achieving high diagnostic accuracy with consistent performance across different Luminex instrumentation platforms, significantly outperforming traditional single-biomarker detection and providing a more reliable tool for non-invasive BCa detection and progression tracking [131]. Most recently, core breakthroughs have focused on in-depth capture of low-abundance proteins and integration of multi-source data. A study constructed a urinary nanoparticle protein corona using Fe_3_O_4_@SiO_2_ nanoparticles and integrated serum and urine proteomic data by combining the transfer learning algorithm (ProteoTransNet) [132]. The constructed diagnostic model achieved an AUC of 0.996 in 1056 multicenter samples, with AUC values of 0.902, 0.926, and 0.941 for staging classification of Ta, T1, and MIBC, respectively. Moreover, stable performance with AUC ≥ 0.98 was maintained in the subgroup of patients with comorbid underlying diseases, representing the latest advancement in proteomic-based BCa detection.

### 4.2. Surface-Enhanced Raman Scattering (SERS) Sensors

SERS is a sensitive, label-free, and molecularly specific non-invasive detection technique, making it a desirable method for BCa diagnosis by enabling single-molecule-level detection of biomolecules in urine.

A serum-based SERS platform has been developed to address the clinical need for non-invasive BCa staging, focusing on distinguishing NMIBC from MIBC [133]. Using gold–silver core–shell nanoparticles as SERS substrates and partial least squares-linear discriminant analysis (PLS-LDA) for spectral data mining, the system achieved 93.3% overall diagnostic accuracy in 90 participants (30 healthy volunteers, 28 NMIBC patients, and 32 MIBC patients). For NMIBC vs. MIBC discrimination, the AUC reached 0.983 with 93.2% accuracy, 90.6% sensitivity, and 96.3% specificity. It identified staging-specific spectral markers: MIBC patients exhibited higher intensities at 494 cm^−1^ (L-arginine), 589 cm^−1^ (amide-VI), 639 cm^−1^ (L-tyrosine), and 1654 cm^−1^ (amide-I/α-Helix) compared to NMIBC, reflecting amino acid metabolism disorder and protein dysregulation in invasive tumors. The platform maintained >88% accuracy in patients with comorbidities, outperforming traditional serum biomarkers such as Carcinoembryonic Antigen (CEA, AUC 0.71).

Using a chemically induced rat model, preclinical validation of SERS for early-stage BCa detection has been conducted, providing key evidence for translational potential [134]. By combining gold nanoparticle SERS substrates with principal component analysis (PCA) and PLS-LDA, this platform analyzed urine samples and achieved an accuracy of 99.6% with an AUC > 0.996 in the diagnosis of early-stage and polyp-form BCa. It detected subtle nucleoside changes (732 cm^−1^, C-N stretching of adenosine) prior to visible tumor formation, thus it identified BCa earlier than cystoscopy, and its spectral signature is highly correlated with human early BCa (r = 0.87)—supporting its relevance for clinical early screening.

To address the issue of underdiagnosis of low-grade BCa, “SERSomes” were introduced, which use multiple individual spectral sets from each sample to comprehensively analyze urine metabolites [135]. In a clinical cohort of 116 participants (31 low-grade BCa T1 patients, 38 low-grade BCa T2 patients, and 47 healthy controls), SERSomes combined with random forest (RF) ML achieved an accuracy of 89.47% (AUC 0.88) for low-grade BCa diagnosis and an accuracy of 90% (AUC 0.83) for T1/T2 stratification. Key contributing spectral bands included 749–767 cm^−1^ (tryptophan/ethanolamine) for diagnosis and 1440–1461 cm^−1^ (deoxyribose/lipid CH_2_ deformation) for stratification. The entire workflow took less than 30 min, enabling a point-of-care (POC) application and avoiding the need for deproteinization pretreatment.

A urine-based SERS sensor using a MXene@gold nanoparticle composite substrate has further expanded clinical applications through the detection of protein biomarkers [136]. The substrate was prepared by hydrothermal synthesis, loaded with 15 nm gold nanoparticles, and modified with anti-BTA monoclonal antibodies. It achieved a BTA detection limit of 0.1 pg/mL, which was 1000-fold lower than that of traditional ELISA. In 210 clinical samples, the sensor could distinguish BCa patients from healthy controls with a sensitivity of 91.7% and a specificity of 93.3%, and differentiate BCa from urinary tract infections with a sensitivity of 89.2% and a specificity of 90.0%, effectively reducing interference from inflammatory factors in the urine matrix. However, it is worth noting that in studies related to blood tests, inflammatory factors may have the potential to predict BC prognosis [137].

With these advances, SERS sensors have evolved from initial detection and early screening to precise solutions for low-grade BCa screening, featuring high-sensitivity, label-free detection, molecular specificity, and minimal invasiveness. These characteristics make them promising for POC testing and large-scale screening applications. Future research will focus on standardizing substrate fabrication and data analysis protocols, integrating SERS with multi-omics techniques, and conducting large-scale prospective trials to accelerate clinical adoption, thereby overcoming current limitations such as matrix interference and inter-study variability.

This sample-preparation-free phase separation and enzyme-responsive POC detection technology has established a new paradigm for the early diagnosis of BCa. The recently developed BLOOM system used hyaluronidase (BCa-specific biomarker) to trigger the degradation of dual hydrogel membranes, releasing encapsulated low-density organic hydrogel signal particles. Driven by buoyancy, these particles autonomously migrated into the oil phase, where they generate solvent-induced fluorescence [138]. This process achieved spatial separation and reading of the signal, thereby completely avoiding interference from complex matrices such as hematuria. The system can directly analyze raw urine samples, combined with a smartphone-based fluorescence detector, at a cost of about $2.5 per test, making it very suitable for home self-testing. In a double-blind validation study with 105 samples, it achieved an AUC of 0.93 for detecting early-stage BCa, with both sensitivity and specificity reaching 88%. By converting enzyme activity into a physical phase-transition signal, this technology enables robust, low-cost POC early screening, representing a significant breakthrough beyond proteomics and SERS.

The development of non-invasive diagnostic technologies for early BCa has progressed from the optimization of traditional ELISA methods to breakthroughs in high-sensitivity detection. Proteomics has improved measurement stability and interference resistance through multi-marker integration, while SERS technology has achieved detection limits at the pg/mL level, addressing the challenge of detecting low-abundance proteins. Notably, novel POC systems based on phase separation and enzyme response, such as the BLOOM system, transform hyaluronidase activity into spatially separated physical signals, enabling high-fidelity detection of raw urine without pretreatment and unaffected by hematuria. This provides a novel approach for low-cost home-based screening. Overall, these advances have increased the detection sensitivity for low-grade tumors from approximately 40% with traditional methods to over 80%, laying a solid foundation for the early diagnosis and precise monitoring of BCa.

## 5. Metabolomic Diagnostic Technologies

Metabolomic-related technologies detect changes in small-molecule metabolites to identify potential diagnostic biomarkers [139], providing new insights for early disease screening and diagnosis. In recent years, various metabolomic detection technologies, such as high-performance liquid chromatography–mass spectrometry (HPLC-MS) and gas chromatography–mass spectrometry (GC-MS), have continuously advanced and been applied in BCa diagnostic research.

### 5.1. High-Performance Liquid Chromatography–Mass Spectrometry (HPLC-MS) Technology

In early studies, researchers employed a combination of a high-performance liquid chromatography system and a hybrid triple quadrupole time-of-flight mass spectrometer for metabolomic analysis of urine samples to distinguish between healthy individuals and BCa patients. The study collected urine samples from 48 healthy individuals and 41 BCa patients, using two statistical methods: PCA and orthogonal partial least squares discriminant analysis (OPLS-DA). Results showed that in positive ionization mass spectrometry mode, OPLS-DA accurately predicted all 48 healthy samples and 41 BCa urine samples [140]. As an unsupervised analysis method, PCA also correctly predicted 46 healthy samples and 40 BCa samples, initially demonstrating the potential of HPLC-MS metabolomic technology for non-invasive early detection of BCa.

With technological advancements, ultra-performance liquid chromatography (UPLC) combined with mass spectrometry has been increasingly applied in urinary metabolomic research on BCa. A study conducted untargeted metabolomic analysis of urine from BCa patients and healthy controls using UPLC-MS to screen for differential metabolites. The study included 57 BCa patients and 38 cancer-free controls, identifying 27 statistically significant differential metabolites (VIP > 1.5 and FDR < 0.05) via PCA and OPLS-DA [141]. This panel of metabolites achieved an AUC of 0.951, sensitivity of 91.2%, specificity of 86.8%, and accuracy of 89.5% in distinguishing BCa patients from cancer-free controls. For differentiating NMIBC from cancer-free controls, the AUC was 0.943, sensitivity 81.6%, specificity 94.6%, and accuracy 88%. Additionally, Pearson correlation coefficient analysis revealed that age, gender, and BMI had weak correlations with the 27 differential metabolites, indicating minimal impact of these factors on the test results. Another study utilized UPLC-MS to analyze urine from 198 BCa patients and 98 healthy volunteers [142]. Results confirmed that p-cresol glucuronide could serve as a diagnostic biomarker for BCa (AUC = 0.79) and had staging value for NMIBC (AUC = 0.803).

**Figure 4:**
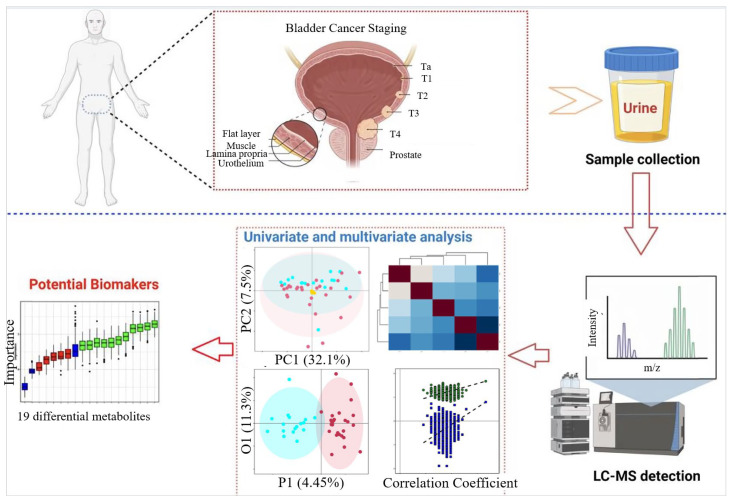
Workflow of urine metabolomics analysis for BCa based on ultra-performance liquid chromatography–mass spectrometry (UPLC-MS). The study identified 19 differential metabolites, primarily associated with metabolic pathways such as phenylacetate metabolism, propanoate metabolism, and fatty acid metabolism. Eleven potential biomarkers were further selected using machine learning approaches, including Random Forest (RF), Support Vector Machine (SVM), and the Boruta algorithm. A logistic regression diagnostic model constructed with these biomarkers demonstrated excellent performance. This workflow underscores its systematic and reliable capacity for discovering metabolic biomarkers with high diagnostic efficacy. Figure reproduced with permission from ref. [143]. Copyright from 2022, Springer Nature Limited.

Furthermore, a study used UPLC-MS for metabolomic analysis of urine from 29 BCa patients and 15 healthy controls, identifying 208 metabolites in total [143], the workflow is shown in Figure 4. OPLS-DA analysis showed significant discriminative potential between the metabolic profiles of BCa patients and healthy controls. And, permutational multivariate analysis of variance validated that the probability of the model occurring randomly was <0.001. Nineteen differential metabolites were screened, mainly involving pathways such as phenylacetate metabolism, propanoate metabolism, and fatty acid metabolism. Eleven potential biomarkers were further selected through Random Forest (RF), support vector machine (SVM), and Boruta analysis, and a logistic regression model was constructed. The ROC curve of this model showed an AUC of 0.983, sensitivity of 95.3%, and specificity of 100%, demonstrating excellent diagnostic performance for BCa.

This technology boasts notable strengths such as high separation efficiency, robust detection sensitivity, and the capacity to identify multiple metabolites simultaneously. Nevertheless, it is not without limitations: the high cost of instrumentation, intricate sample pretreatment procedures, and prolonged detection cycles collectively hinder its wide-spread implementation in primary medical institutions.

### 5.2. Gas Chromatography–Mass Spectrometry (GC-MS) Technology

Due to the high-resolution advantage of gas chromatography-time-of-flight mass spectrometry (GC×GC TOF MS), it has been used to detect urinary volatile organic compounds (VOCs) for identifying diagnostic biomarkers of BCa. A study analyzed 218 VOCs in urine from 75 BCa patients (48 NMIBC, 27 MIBC) and 50 healthy controls using headspace solid-phase microextraction (HS-SPME) combined with two-dimensional GC×GC TOF MS. Orthogonal partial least squares discriminant analysis (OPLS-DA) screened 12 significantly different VOCs, among which six metabolites (2-methoxyphenol, phenylacetaldehyde, nonanal, decanal, isovaleric acid, and phenylpropionic acid) were detected only in the urine of BCa patients. A random forest diagnostic model built on these 12 VOCs achieved a sensitivity of 89% and specificity of 94% in the training set (80% of samples) and a sensitivity of 85% and specificity of 91% in the validation set (20% of samples). Additionally, the model’s sensitivity for MIBC (92.6%) was slightly higher than that for NMIBC (82.3%) [144].

A subsequent multicenter cohort study validated urinary VOCs in 230 participants (110 BCa patients, 120 healthy controls), confirming eight previously identified differential VOCs (including 2-methoxyphenol and nonanal) with consistent expression patterns. A logistic regression model based on these VOCs achieved an AUC of 0.77, sensitivity of 71%, and specificity of 72% in external validation. Meanwhile, it was found that the VOC profile remained stable within 48 h after urine collection, providing a basis for standardized sample processing [144]. Another detection protocol constructed with six VOCs of statistically significant abundance achieved an AUC of 0.80, sensitivity of 71%, and specificity of 80%, offering a new non-invasive tool for BCa diagnosis and surveillance [144]. This technology excels in detecting volatile metabolites with high separation efficiency. However, it has drawbacks such as difficulty in detecting non-volatile metabolites, cumbersome sample pretreatment, long detection times, and poor result reproducibility easily affected by experimental conditions.

### 5.3. Gas Chromatography–Ion Mobility Spectrometry (GC-IMS) Technology

GC-IMS has emerged as a crucial technology for rapid urinary screening of BCa, leveraging the combined advantages of chromatographic separation capability and high ion mobility spectrometry sensitivity, with a typical detection time of less than 30 min [145,146]. Its features of no complex sample pretreatment, simple operation, and portability endow it with significant application potential in clinical screening scenarios [146].

A clinical study involving 106 subjects (including 15 BCa patients, 55 prostate cancer patients, and 36 non-cancer controls) utilized a GC-IMS device to analyze VOCs in urine headspace, with a detection time of only 10 min [145]. Through data extraction via VOCal software, 10-fold cross-validation, and analysis combining models such as logistic regression and random forest, the technology exhibited excellent performance in distinguishing BCa from non-cancer populations, achieving an AUC of 0.95, a sensitivity of 87%, and a specificity of 92%. When differentiating BCa from prostate cancer, the AUC reached as high as 0.97, demonstrating strong tumor-specific discriminative ability [145]. The study also identified 13 BCa-specific VOC biomarkers, including biphenyl, nonanal, and tetradecane, which were validated by PubChem and NIST databases, providing a molecular basis for the accurate diagnosis of BCa [145].

Another multicenter study involving 115 urine samples adopted a rapid GC-IMS analytical method without sample pretreatment (detection time: 10 min) and successfully identified 23 VOCs, covering 7 categories such as ketones, aldehydes, alcohols, and sulfur compounds [146]. Among them, acetone and 2-butanone were detected in all samples, and concentration changes of compounds such as 2-pentanone and hexanal were closely related to pathological states like BCa [146]. The technology achieved a low limit of detection (LOD) of 4.66 ppb and maintained a good linear relationship within the concentration range of 0-128 ppb. Its analytical results were highly consistent with traditional GC-MS technology, confirming the reliability of its detection [146]. Additionally, analysis of subgroup samples with excessive ketone bodies (>60 mg/dL) showed that VOCs such as 3-methylbutanal and pentanol could serve as specific indicators, offering new ideas for the screening of BCa patients with metabolic abnormalities [146].

A systematic review including 1266 subjects further verified the value of GC-IMS-related technologies in BCa diagnosis [147]. The review pointed out that urinary VOC analysis, as a non-invasive diagnostic method, shows promising prospects in early cancer detection. Among them, GC-IMS technology can effectively capture differences in BCa-related VOC fingerprint spectra through a dual-physical separation mechanism [147]. Despite limitations such as sample size heterogeneity and inconsistent detection standards in existing studies, the advantages of GC-IMS in detection speed and operational simplicity make it particularly suitable for large-scale screening of high-risk populations in community hospitals and primary medical institutions [145,147]. The core advantages of this technology include rapid detection (10–15 min per sample), no need for complex sample pretreatment, portable equipment, and low detection limits [145,146]. However, it also has limitations: compared with GC-MS, its resolution is relatively low, the detection sensitivity for some low-concentration metabolites is insufficient, and the specificity of biomarkers needs further optimization through multicenter large-sample studies [146,147]. In the future, standardizing detection procedures, expanding sample cohorts, and integrating multi-omics analysis are expected to further enhance its clinical application value in early BCa screening and recurrence monitoring.

Significant progress has been made in urine-based metabolomics diagnostic techniques for early BCa. From the early HPLC-MS and GC-MS technologies to the subsequent GC-IMS technology, each method has its unique characteristics and advantages. By screening differential metabolites and building diagnostic models, these technologies have shown good potential in the diagnosis of early BCa. Some technologies have been validated in multicenter studies, significantly improving the detection rate of early BCa. Nevertheless, current diagnostic technologies still face several challenges, including high instrument costs, complex sample pretreatment, insufficient specificity of biomarkers, and unstable detection results. To promote the development of these technologies toward standardization and portability, thereby enabling their widespread application in clinical diagnosis of early BCa, future efforts should focus on further optimizing technical parameters, reducing costs, improving detection specificity and sensitivity, and conducting larger-scale clinical validation.

## 6. Conclusions and Outlook

To facilitate the comparison of key diagnostic technologies for BCa and enhance clinical applicability, a concise summary table of five core diagnostic approaches is shown in Table 1. For the non-invasive urine-based diagnosis of early BCa, techniques have progressed from traditional cytology, which relies on morphological observation, to molecular diagnosis based on multi-omics biomarkers. With DL, AI has significantly enhanced the automation and accuracy of urine cytology image analysis. Genomic technologies, including PCR and NGS, can detect DNA mutations, copy number variations, and methylation patterns with high-throughput and high sensitivity. Transcriptomics (mRNA, miRNA, and lncRNA) and proteomics/metabolomics further reveal the characteristic molecular profiles of tumors from a functional perspective. Overall, these advances have increased the detection sensitivity of low-grade, early-stage tumors from less than 50% with conventional methods to over 80%, and the AUC values of some techniques even exceed 0.95, thereby laying a solid foundation for molecular-based diagnosis of early BCa.

However, key challenges still remain. First, the lack of standardization and clinical validation leads to high heterogeneity in test results due to differences between various platforms and operational procedures. Therefore, large-scale, prospective multicenter studies are needed to establish unified standards. Second, there are still limitations in detecting early, tiny tumors. The abundance and specificity of existing biomarkers in low tumor load or in situ cancer lesions needs further improvement. Third, there is a trade-off between cost and performance. The high cost of high-sensitivity technologies (e.g., WGS, deep proteomics) hinders their application in primary care and population screening.

To address the current limitations, future development is expected to focus on the following four directions. Integration of multi-omics combined with AI-driven analysis: By combining genomic, epigenomic, proteomic, and metabolomic data, multi-dimensional diagnostic models can be constructed, and AI algorithms can be used to identify deep-relevant features, achieving more precise risk stratification and early detection of BCa. Innovation of portable POC and home testing devices: Efforts should be made to continuously develop portable devices based on phase-separation sensing (e.g., the BLOOM system), microfluidic chips, and smartphone-compatible readers to promote real-time, convenient, and low-cost testing, empowering home health management. Application of liquid biopsy for dynamic monitoring: Further apply urine-based liquid biopsies (e.g., cfDNA and exosomes) to monitor molecular residual disease and clonal evolution during treatment, providing dynamic molecular evidence for evaluating treatment response and early warning of recurrence. Finally, to address the core bottleneck of “lack of standardization” in the current field, unified standardized implementation protocols for multicenter trials are formulated and large-scale validations are conducted: (1) Standardized sample collection and preservation—unify urine sample collection containers, preservation temperatures (4 °C for short-term storage, −80 °C for long-term storage), and storage durations (no more than 24 h for unprocessed samples) to avoid degradation of biomarkers such as nucleic acids and proteins. (2) Unified technical specifications for detection platforms—for core technologies including AI-based cytology analysis, NGS, and SERS, establish standardized operation procedures (SOPs) for instrument parameters, reagent types, and data analysis algorithms to ensure consistency of detection results across different laboratories. (3) Standardized result interpretation and reporting—develop unified diagnostic threshold criteria for different biomarkers (e.g., methylation levels, miRNA expression abundance) and a standardized reporting format including key indicators such as sensitivity, specificity, and predictive values, to facilitate clinical interpretation and cross-study comparison. (4) Standardized design and implementation of multicenter trials—adopt a unified trial protocol including inclusion/exclusion criteria for participants, sample size calculation methods, and statistical analysis plans; entrust data management and statistical verification to an independent third-party institution to ensure the objectivity and authenticity of trial results. These standardized protocols will effectively reduce technical heterogeneity, enhance the credibility and reproducibility of research findings, and accelerate the translational application of non-invasive urine-based diagnostic technologies in clinical practice.

In summary, through the convergence of multiple disciplines and integration of various technologies, non-invasive urine-based diagnostic methods are gradually becoming an essential component of the early screening, precise diagnosis, and comprehensive management system for BCa. This brings the possibility of fundamentally improving patient outcomes and alleviating the societal healthcare burden.

## Figures and Tables

**Table 1:** Non-invasive urine-based diagnostic technologies for early bladder cancer.

Technology Category	Core Detection Target/Principle	Key Advantages	Limitations	Clinical Role (Screening/Monitoring)	Refs.
Cystoscopy Combined with Tissue Biopsy	Direct visualization of bladder mucosa; pathological confirmation of suspicious lesions via biopsy	Gold standard for confirming BCa diagnosis; high diagnostic reliability	Invasive; potential complications (e.g., hematuria, infection); high cost; patient discomfort	Monitoring (high-risk/post-treatment patients)	[10]
Conventional Urine Cytology	Microscopic examination of urinary cells to identify malignant or suspicious morphological features	Non-invasive; simple operation; low-cost; specificity >90%	Low sensitivity (≈42% for early/low-grade tumors); high subjectivity; inconsistent protocols across institutions	Preliminary screening; auxiliary monitoring (post-treatment)	[10,13]
AI-based Urine Cytological Detection Technology	Automated classification of benign/malignant cells by analyzing urine cytological morphology (nuclear–cytoplasmic ratio, chromatin abnormalities, etc.) using CNN and other algorithms	Non-invasive; reduces subjectivity; improves sensitivity for low-grade tumors (VisioCyt reaches 77%); high diagnostic efficiency	Lack of standardized training/validation protocols; reliance on high-quality image datasets	Screening + Monitoring (postoperative recurrence)	[30,31,32,33,34,35,36,37,38,43,44,45,47,48]
Urine Genomic Detection Technology	1. DNA level: Gene mutations (TERT, FGFR3), Copy Number Variations (CNVs) (PCR/NGS); 2. Methylation: Methylation status of target genes (MSP, WGBS)	Non-invasive; high specificity; capable of detecting early micro-tumors; Uromonitor^®^ has a Negative Predictive Value (NPV) of 98.8%	High detection cost; complex experimental procedures; requires professional technical support	Screening (high-risk populations) + Monitoring (recurrence)	[59,61,63,64,74,75,85,89]
Urine RNA Detection Technology	mRNA (ETS2/uPA, IGF2), miRNA (miR-21-5p, 6-miRNA signature), lncRNA (UCA1, uc004cox.4)	Stable biomarkers; multi-gene combination improves accuracy; some technologies enable rapid detection (within 30 min)	Lack of standardization in sample processing and detection platforms; insufficient enrichment efficiency for extremely early micro-tumors	Screening + Monitoring (prognostic stratification)	[108,111,115,124,125]
Proteomic Detection Technology	Targeted detection of specific urinary proteins (BTA, NMP22), peptides; single-molecule level detection by SERS; integrated analysis of multi-protein panels	Non-invasive; simple and rapid operation; SERS achieves a detection limit of 0.1 pg/mL; low-cost of Point-of-Care (POC) systems ($2.5 per test)	Low specificity; susceptible to interference from benign urological diseases (infections, stones); some technologies rely on high-end equipment	Screening (primary care) + Monitoring (treatment efficacy)	[132,134,138]
Metabolomic Diagnostic Technology	Detection of differential urinary metabolites (VOCs, p-cresol glucuronide, etc.) based on HPLC-MS/GC-IMS and other technologies	Non-invasive; real-time reflection of tumor metabolic status; rapid detection by GC-IMS (<30 min)	Lack of standardized detection protocols; susceptible to interference from diet and medications; insufficient large-scale clinical validation	Screening (high-risk populations) + Monitoring (recurrence)	[143,145,147]

## Data Availability

No new data were created or analyzed in this study. Data sharing is not applicable to this article.

## Abbreviations

The following abbreviations are used in this manuscript:

AI	Artificial Intelligence
ML	Machine Learning
DL	Deep Learning
ANN	Artificial Neural Network
CNN	Convolutional Neural Network
BCa	Bladder Cancer
NMIBC	Non-Muscle Invasive Bladder Cancer
MIBC	Muscle Invasive Bladder Cancer
UTUC	Upper Tract Urothelial Carcinoma
PCR	Polymerase Chain Reaction
qPCR	Quantitative Polymerase Chain Reaction
NGS	Next-Generation Sequencing
WGS	Whole-Genome Sequencing
sWGS	Shallow Whole-Genome Sequencing
LC-WGS	Low-Coverage Whole-Genome Sequencing
CNV	Copy Number Variation
MSP	Methylation-Specific PCR
ELISA	Enzyme-Linked Immunosorbent Assigmnt
SERS	Surface-Enhanced Raman Scattering
HPLC-MS	High-Performance Liquid Chromatography–Mass Spectrometry
GC-MS	Gas Chromatography–Mass Spectrometry
GC-IMS	Gas Chromatography–Ion Mobility Spectrometry
DNA	Deoxyribonucleic Acid
RNA	Ribonucleic Acid
mRNA	Messenger RNA
miRNA	MicroRNA
lncRNA	Long Non-Coding RNA
cfDNA	Cell-Free DNA
AUC	Area Under the Curve
NPV	Negative Predictive Value
PPV	Positive Predictive Value
ROC	Receiver Operating Characteristic
EV	Extracellular Vesicle
POC	Point-of-Care
